# Covid Adult Mortality in Brazil: An Analysis of Multiple Causes of Death

**DOI:** 10.3389/fpubh.2021.788932

**Published:** 2022-01-17

**Authors:** Ana Maria Nogales Vasconcelos, Lenice Ishitani, Daisy Maria Xavier Abreu, Elisabeth França

**Affiliations:** ^1^Department of Statistics, University of Brasilia, Brasilia, Brazil; ^2^Epidemiology and Health Assessment Research Group (GPEAS), Federal University of Minas Gerais, Belo Horizonte, Brazil; ^3^Graduate Program in Public Health, School of Medicine and Epidemiology and Health Assessment Research Group (GPEAS), Federal University of Minas Gerais, Belo Horizonte, Brazil

**Keywords:** COVID-19, multiple cause of death, non-communicable diseases, Brazil, mortality

## Abstract

**Objective:**

This study aimed to analyze the chain of events and contributing causes associated with COVID-19 adult mortality (30–69 years old), based on qualified data on CoD from three Brazilian capitals cities, Belo Horizonte, Salvador, and Natal, in 2020.

**Methods:**

Data of all deaths among residents in the three capitals in 2020 were provided by these municipalities' routine Mortality Information System (SIM). Mentions B34.2 with the markers U07.1 and U07.2 in the death certificate identified COVID-19 deaths. We used a multiple-cause-of-death approach better to understand the complexity of the morbid process of COVID-19. Conditions that appeared more frequently in the same line or above the COVID-19 mentions in the death certificate were considered a chain-of-event. Conditions that occurred more often after the codes for COVID-19 were considered as contributing.

**Results:**

In 2020, 7,029 records from COVID-19 as the underlying cause of death were registered in SIM in the three capitals. Among these, 2,921 (41.6%) were deceased between 30 and 69 years old, representing 17.0% of deaths in this age group. As chain-of-events, the most frequent conditions mentioned were sepsis (33.4%), SARS (32.0%), acute respiratory failure (31.9%), unspecified lower respiratory infections (unspecified pneumonia) (20.1%), and other specified respiratory disorders (14.1%). Hypertension (33.3%), diabetes unspecified type (21.7%), renal failure (12.7%), obesity (9.8%), other chronic kidney diseases (4.9%), and diabetes mellitus type 2 (4.7%) were the most frequent contributing conditions. On average, 3.04 conditions were mentioned in the death certificate besides COVID-19. This average varied according to age, place of death, and capital.

**Conclusion:**

The multiple-cause analysis is a powerful tool to better understand the morbid process due to COVID-19 and highlight the importance of chronic non-communicable diseases as contributing conditions.

## Introduction

The importance of vital statistics to support the planning, evaluation, and monitoring of health programs and policies is widely recognized (1, 2). In the current scenario of the COVID-19 pandemic, the availability of vital statistics data is essential to follow the evolution and characteristics of cases and deaths (3). This information must be timely and with a satisfactory level of quality for its use. In Brazil, the Mortality Information System (SIM) from the Ministry of Health (MS) provides data on mortality for the whole country since 1976. SIM is a decentralized system in which each municipality (5,570 in total) is responsible for collecting and inputting data from the death certificate that the physician must sign (4). Although recent studies have shown that SIM has a high completeness level, there are still problems with the quality of information on causes of death (CoD) (5).

Several initiatives to improve the quality of information have been adopted, including physician training to improve the certification of the cause of death. The investigation of deaths with underlying cause classified as garbage code (CG) was carried out in 2017 in 60 municipalities in the five regions of the country, an initiative coordinated by the MS, in partnership with the Federal University of Minas Gerais (UFMG) and with support from Vital Strategies, through the Bloomberg Foundation. The investigation resulted in the reclassification for specific causes of 58% of deaths with GC (6).

In 2019, to promote the sustainability of the previous improvements achieved in selected municipalities in Brazil, a new initiative proposed to work more deeply with only three cities, Belo Horizonte with 2.5 million inhabitants, Salvador, 2.9, and Natal, 0.9 (7). The first municipality is in the Southeast region and the two others in the Northeast. Due to the emergence of the new Coronavirus (COVID-19), the initiative in these three cities changed its former aim and focused on an investigation to correctly identify deaths from COVID-19.

COVID-19 epidemic has led to an overload on the health system in Brazil, with a negative impact on all health programs (8). It has significantly compromised the achievement of Goal 3 of the Sustainable Development Objectives (SDG), more specifically, the target 3.4 of SDG that pointed, by 2030, a reduction of one-third premature mortality (adults under 70 years old) from non-communicable diseases (NCD) (9).

Non-communicable diseases are the leading causes of death (CoD) for adults and the elderly (10). They are also the main comorbidities that contribute to the severity of COVID-19 (11). As death is not a single event, the multiple-cause-of-death approach can contribute to understanding the complexity of the morbid process of COVID-19 since the traditional focus on underlying CoD statistics is insufficient in facing the challenges posed by this epidemic (12).

To better describe and understand the morbid process of COVID-19 mortality and its association with NCD, the present study aims to analyze the chain of events and contributing causes associated with COVID-19 adult mortality (30–69 years old), based on qualified data on CoD from the three capitals cities in 2020. Although these three capitals do not represent the entire country, they can provide essential information about the morbid process caused by COVID-19 and its contributing causes.

## Materials and Methods

### Data

We used data of all deaths among residents in the three capitals (Belo Horizonte, Salvador, and Natal) that occurred in 2020, provided by these municipalities' routine Mortality Information System (SIM). The available database contains information on the characteristics of the deceased, the circumstance of death, causes of death (underlying cause and associated causes), and the investigation process. For this study we selected deaths from 30 to 69 years old that correspond to premature mortality, according to the WHO Global Action Plan for the prevention and control of non-communicable diseases (13, 14).

### Cause of Death Statistics

Even though to statistical purpose, for each death, only one CoD is tabulated, certifier physicians must fill the death certificate with all conditions considered to have caused or contributed to the death. The order that all causes are mentioned in the death certificate can show the sequence of events that caused the death. In that sequence, the underlying cause of death (UCoD) will be (a) the disease or injury that initiated the train of events leading directly to death, or (b) the circumstances of the accident or violence that produced the fatal injury.

This sequence results from a complex pathological process. Thus, the UCoD approach underestimates mortality from diseases not selected as the underlying cause but contributing to death, as with chronic conditions. Using the multiple-cause-of-death approach, all causes present in the morbid process and informed by the certifying physician can be analyzed.

### Medical Certification and ICD Mortality Coding of COVID-19

The first step in this work was to understand the medical certification and ICD mortality coding of COVID-19 in Brazil. For the codification of the COVID-19 death, Ministry of Health guidelines (15) established the use of ICD-10 code B34.2 with markers U07.1 (COVID-19, virus identified) and U07.2 (COVID-19, virus not identified). The markers would help the investigation of the cause of death process, specifying if the diagnosis of COVID-19 was confirmed or not. At the beginning of the pandemic, coders used the U04.9 code that refers to severe acute respiratory syndrome (SARS) to identify deaths from COVID-19 in the country. In July 2020, the MH issued a note informing that the codification of deaths with a mention of COVID-19 should be revised (16). The U04.9 code would only remain in the SIM if the SARS was part of the events chain that led to death.

Then, to identify the deaths due to COVID-19, we considered the mentions B34.2 with the markers U07.1 and U07.2 in the death certificate.

### Multiple Cause of Death Analysis

To proceed with the analysis of causes associated with COVID-19 mortality, we used a multiple-cause-of-death approach, including all conditions, diseases, and injuries mentioned on the death certificate. Considering the significant number of possible conditions informed in the DC for this analysis, we decided to group the ICD-10 codes into groups with similar diagnoses. The initial classification was based on the Global Burden of Diseases study (GBD 2017) (17) and adapted to the Brazilian epidemiological profile. The Supplementary Table A presents the Group of conditions analyzed and respective ICD-10 codes.

We classified the conditions into two categories: chain-of-event or contributing. In Part I of the death certificate, we considered conditions that appeared more frequently (60% or more) in the same line or above the mentions B34.2, U0.7.1, U07.2, as a chain-of-event of COVID-19 mortality. Conditions that appeared more frequently (60% or more) after the codes for COVID-19 in Part I or, mainly, in Part II, were considered a contributing condition. Conditions that did not meet the requirements above or mentioned in <0.5% of the analyzed death certificates were not classified.

### Statistical Analysis

Other than the cause of death, we consider age, gender, and race/color as characteristics of the deceased and local of death.

All data were analyzed using R-Studio with libraries readxl, foreign, tidyverse, and writexl.

### Ethics

The analyzed data were in the public domain, preserving anonymity. Therefore, the project was approved by the Research Ethics Committee of the Federal University of Minas Gerais (COEP/UFMG) under number 4749326.

## Results

**Figure 1:**
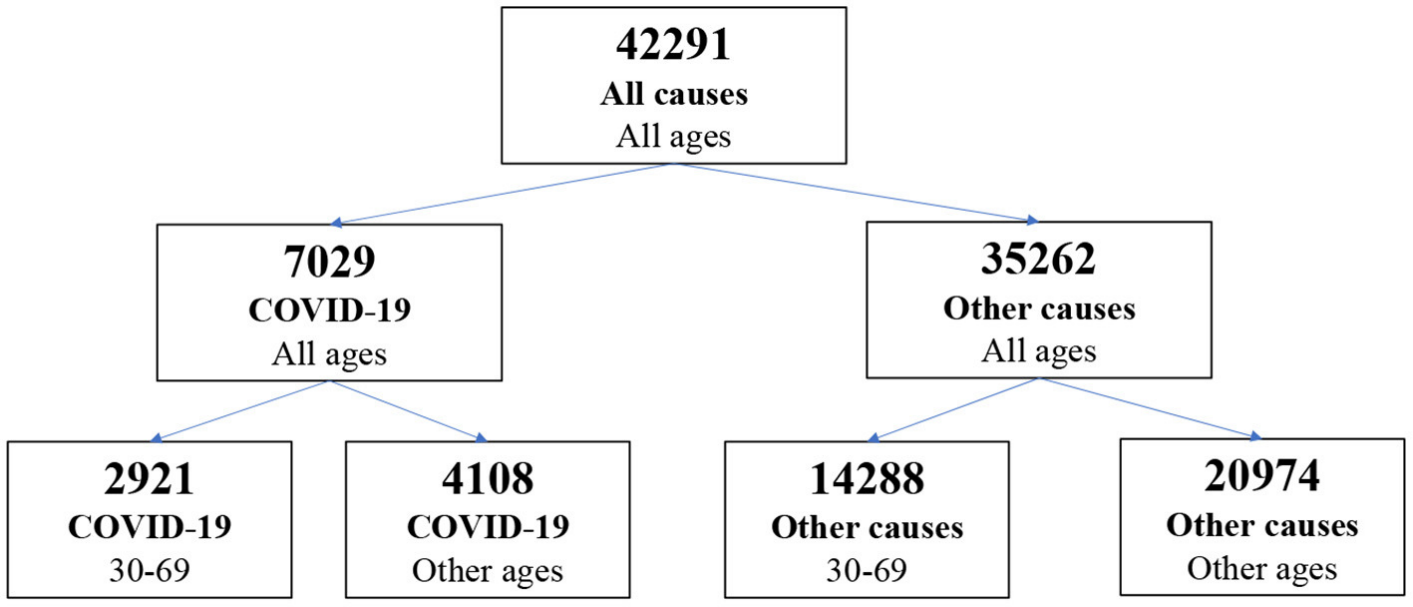
Number of death certificates according to age and cause of death, in three Brazilian capitals, 2020.

In 2020, 7,029 records from COVID-19 as the underlying cause of death (UCoD) were registered by the routine SIM in the three Brazilian capitals cities (Belo Horizonte, Salvador, and Natal) (Figure 1). Of these, 62.5% were investigated to confirm the COVID-19 diagnosis and complete information about causes of death. The three capitals presented different investigation rates for COVID-19 mortality: 93.4% in Belo Horizonte (*n* = 2,018), 48.2% in Salvador (*n* = 1,812), and 51.9% in Natal (*n* = 571).

Among the deaths from COVID-19 as UCoD, 2,921 (41.6%) were from deceased between 30 and 69 years old, representing 17.0% of deaths in this group of ages. The virus SARS-COV2 was identified in 85.8% of deaths (U07.1), and for 14%, the COVID-19 diagnosis was clinical or epidemiological (U07.2).

Table 1 shows the distribution of COVID-19 deaths according to some characteristics and the proportion in relation to the total number of deaths in each category. Firstly, the number of deaths is concentrated in older ages: 50.2% of deaths are in the 60–69 age group. Likewise, the proportion of COVID-19 deaths increases with age: from 11% (IC 95% 9.5–12.4%) in the 30–39 age group to 19.2% (IC 95% 18.3–20.1%) in the 60–69 age group.

**Table 1:** Number of death certificates due to COVID-19 and all causes of death according to characteristics of the deceased and local of death, at ages 30–69 years, in three Brazilian capitals, 2020.

**Characteristics**	**Number of death certificates**			**Ratio**		
	**All causes**	**COVID-19**		**(COVID-19/All causes)**		
		***n***	**%**	**%**	**IC 95%**	
Total	17,209	2,921	100.0	17.0	16.4	17.5
**Age**						
30–39	1,716	188	6.4	11.0	9.5	12.4
40–49	2,815	408	14.0	14.5	13.2	15.8
50–59	5,045	860	29.4	17.0	16.0	18.1
60–69	7,633	1,465	50.2	19.2	18.3	20.1
**Gender**						
Female	7,035	1,205	41.3	17.1	16.2	18.0
Male	10,174	1,716	58.7	16.9	16.1	17.6
**Race/color**						
White	4,675	731	25.0	15.6	14.6	16.7
Black	3,173	585	20.0	18.4	17.1	19.8
Brown	8,654	1,407	48.2	16.3	15.5	17.0
Others	707	198	6.8	28.0	24.7	31.3
**Place of death**						
Outside a hospital or health center	4,173	121	4.1	2.9	2.4	3.4
Inside a hospital or health center	13,036	2,800	95.9	21.5	20.8	22.2
**Capital**						
Belo Horizonte	6,810	790	27.0	11.6	10.8	12.4
Salvador	7,916	1,686	57.7	21.3	20.4	22.2
Natal	2,483	445	15.2	17.9	16.4	19.4

*Source: Ministry of Health, Mortality System Information*.

Although the number of deaths from COVID-19 is higher among men (58.7%), the proportion of deaths from COVID-19 in the total number of deaths is very similar between men and women, between 30 and 69 years (17.1 among women and 16.9 among men).

Regarding race/color categories, 68.2% of death from COVID-19 are of blacks or browns in these three capitals. We can also observe that the proportion of COVID-19 deaths among whites (15.6–IC 95% 14.3–16.7%) is lower than among blacks (18.4–IC 95% 17.1–19.8%). Among the “others” category of race/color, the proportion of deaths by COVID-19 is much higher (25.5–IC 95% 24.7–31.3%).

Concerning the place of occurrence of the death, we found that 95.9% of deaths occurred in hospitals or health centers. Thus, the proportion of COVID-19 deaths in total hospital deaths is much higher than that among deaths occurring outside hospital units (21.5 vs. 2.9%).

Considering the distribution of deaths among the three capitals, we observed that Salvador concentrated 57.7% of COVID-19 deaths in 2020. The proportion of COVID-19 deaths in the total number of deaths in Salvador is much higher than in the other two capitals [21.3% (20.4–22.2%) in Salvador vs. 17.9% (16.4–19.4%) and 11.6% (10.8–12.4%) in Natal and Belo Horizonte, respectively].

Table 2 shows the conditions mentioned in at least 0.5% of the death certificates where COVID-19 was the UCoD, and their classification as chain-of-events, contributing condition or not classified. Supplementary Table B presents all conditions mentioned classified as chain-of-events and contributing conditions. In 98.4% of DC with COVID-19 as UCoD (*n* = 2,843), there was at least one condition mention besides the COVID-19 diagnosis.

**Table 2:** Most frequent conditions listed in death certificates by position in relation to COVID-19 diagnosis, at ages 30–69 years, in three Brazilian capitals, 2020.

**Conditions (group of IC-10 codes)**	**Number of mentions**		**Position in relation to COVID-19 diagnosis**		**% of COVID-19 deaths certificates (*n* = 2,843)**
	***n***	**%**	**Above**	**Below**	
**Chain-of-events**					
Sepsis	950	10.8	96.1	3.9	33.4
SARS	909	10.3	99.4	0.6	32.0
Acute respiratory failure	907	10.3	96.1	3.9	31.9
Unspecified lower respiratory infectious (Unspecified Pneumonia)	571	6.5	94.2	5.8	20.1
Other specified respiratory disorders (SARS*)	400	4.5	96.5	3.5	14.1
Cardiac arrest and shock	257	2.9	97.7	2.3	9.0
Other lower respiratory infections (Pneumonia)	141	1.6	95.0	5.0	5.0
Other respiratory diseases	105	1.2	94.3	5.7	3.7
Symptoms and signs not classified elsewhere	98	1.0	96.7	3.3	3.4
Asphyxia and hypoxemia	89	1.0	94.4	5.6	3.1
Pulmonary embolism	61	0.7	77.0	23.0	2.1
External causes–other factors	30	0.3	86.7	13.3	1.1
**Contributing conditions**					
Hypertension	947	10.8	0.4	99.6	33.3
Diabetes unspecified type	617	7.0	0.5	99.5	21.7
Renal failure	360	4.1	30.3	69.7	12.7
Obesity	280	3.2	0.0	100.0	9.8
Other chronic kidney diseases	140	1.6	13.6	86.4	4.9
Diabetes mellitus type 2	133	1.5	0.8	99.2	4.7
Left heart failure	104	1.2	26.0	74.0	3.7
Ischemic heart disease	100	1.1	38.0	62.0	3.5
Hemodialysis	83	0.9	21.7	78.3	2.9
Chronic obstructive pulmonary disease	79	0.9	5.1	94.9	2.8
Chronic kidney disease due to glomerulonephritis	79	0.9	7.6	92.4	2.8
Other mental disorders	65	0.7	1.5	98.5	2.3
Unspecified stroke	62	0.7	17.7	82.3	2.2
Alcohol use disorders	42	0.5	0.0	100.0	1.5
Adverse effects of medical treatment	42	0.5	38.1	61.9	1.5
Asthma	41	0.5	4.9	95.1	1.4
Cirrhosis and other chronic liver diseases	35	0.4	8.6	91.4	1.2
Tracheal, bronchus, and lung cancer	33	0.4	3.0	97.0	1.2

*Source: Ministry of Health, Mortality System Information*.

As chain-of-events, the most frequent conditions mentioned on the death certificate were sepsis (33.4%), SARS (32.0%), acute respiratory failure (31.9%), unspecified lower respiratory infections (unspecified pneumonia) (20.1%), and other specified respiratory disorders (14.1%). These five conditions corresponded to 42.4% of all conditions mentioned in DC analyzed.

Among the other conditions in the chain of events, we highlight the external cause–other factors condition mentioned in 1.1% of DC. A more detailed analysis showed that the most frequent code in this group (77%) corresponds to Y95, nosocomial condition.

As contributing conditions, hypertension (33.3%), diabetes unspecified type (21.7%), renal failure (12.7%), obesity (9.8%), other chronic kidney diseases (4.9%), and diabetes mellitus type 2 (4.7%) were the most frequent conditions mentioned in DC. These six conditions corresponded to 28.1% of all conditions mentioned in DC analyzed.

Among the other contributing conditions, we have mentions related to cardiovascular diseases, as left heart failure (3.7%), ischemic heart disease (3.5%), and unspecified stroke (1.5%), related to kidney diseases, as hemodialysis (2.9%) and chronic kidney disease due to glomerulonephritis (2.8%), related to respiratory diseases, as chronic obstructive pulmonary disease (2.8%) and asthma (1.4%), related to mental disorders, as alcohol use disorders (1.5%) and other mental disorders (2.3%). Related to neoplasms, the condition most frequent was tracheal, bronchus, and lung cancer (1.2%).

Still, we have external causes as the adverse effects of medical treatment in 1.5% of DC as contributing conditions.

Conditions not classified as chain-of-events or contributing were the group of injuries (0.8%), gastrointestinal bleeding (0.8%), flutter and fibrillation (0.7%), and hepatic failure (0.7%), among others less frequently mentioned.

Table 3 shows the distribution of death certificates according to the type of conditions associated with COVID-19. In general, 64.1% of DC present the two kinds of conditions: chain-of-events and contributing. This proportion varies according to (1) the age, from 51.6% in the 30–39 age group to 66.1% in the 60–69; (2) the gender, 67.8% among women and 61.5% among men; (3) the place of death, 64.4% for deaths occurred inside a hospital or health center, and 56.2% for deaths occurred elsewhere; (4) the capitals, 64.1 and 66.2% in Belo Horizonte and Salvador, against 56.2% in Natal.

**Table 3:** Distribution of conditions associated with COVID-19 by category according to characteristics of the deceased and local of death, at ages 30–69 years, in three Brazilian capitals, 2020.

**Characteristics**	**Number of death certificates**	**Category of condition associated with COVID-19 (%)**				
		**None**	**Chain of events only**	**Contributing only**	**Chain of events and contributing**	**Not classified**
Total	2,921	2.7	25.2	7.6	64.1	0.4
**Age**						
30–39	188	2.7	36.7	8.0	51.6	1.1
40–49	408	2.0	28.2	7.1	62.3	0.5
50–59	860	2.7	24.9	8.0	64.2	0.2
60–69	1,465	2.9	23.1	7.4	66.1	0.4
**Gender**						
Female	1,205	1.7	22.7	7.1	67.8	0.7
Male	1,716	3.3	27.0	7.9	61.5	0.2
**Race/color**						
White	731	2.3	26.3	8.1	62.9	0.4
Black	585	3.1	25.3	8.9	62.2	0.5
Brown	1,407	2.6	24.6	7.2	65.2	0.4
Others	198	3.0	25.8	5.1	66.2	0.0
**Place of death**						
Outside a hospital or health center	121	7.4	19.8	16.5	56.2	0.0
Inside a hospital or health center	2,800	2.5	25.5	7.2	64.4	0.4
**Capital**						
Belo Horizonte	790	1.3	26.6	7.5	64.1	0.6
Salvador	1,686	2.8	25.0	5.7	66.2	0.4
Natal	445	4.7	23.8	15.1	56.2	0.2

*Source: Ministry of Health, Mortality System Information*.

We can note that deaths that occurred outside a hospital or health center are more likely to have no other condition mentioned in DC besides COVID-19 (7.4%) or contributing only (16.5%). We observe the same for deaths registered in the city of Natal. We also note that deaths in the 30–39 age group are more likely to present only chain-of-events conditions (36.7%) than other groups of ages. We observe the same for men (27.0%) in comparison to women (22.7%).

For all death certificates analyzed, we have 3.04 (IC 95%: 2.99–3.10) conditions mentioned on average in Table 4. Nevertheless, this average varied according to age, place of death, and capital. For the first characteristic, we observe that the mean number of conditions increases with age. From 2.48 mentions (IC 95%: 2.30–2.66) on average for the age group 30–39 to 3.16 mentions (IC 95%: 3.08–3.24) for 60–69. Deaths that occurred inside a hospital or health center present a higher number of conditions mentioned on average than deaths that occurred elsewhere (3.07 vs. 2.53). Belo Horizonte presents a higher number of conditions mentioned on average than the other two capitals. In Belo Horizonte, the mean number of conditions mentioned was 3.39 (IC 95%: 3.28–3.50) against 2.94 (IC 95%: 2.87–3.01) and 2.81 (IC 95%: 2.68–2.94) for Salvador and Natal, respectively.

**Table 4:** Mean number of conditions mentioned in death certificate according to characteristics of the deceased and local of death, at ages 30–69 years, in three Brazilian capitals, 2020.

**Characteristics**	**Mean of conditions (besides COVID-19)**		
	**Mean**	**IC 95%**	
Total	3.04	2.99	3.10
**Age**			
30–39	2.48	2.30	2.66
40–49	2.85	2.72	2.98
50–59	3.07	2.98	3.16
60–69	3.16	3.08	3.24
**Gender**			
Female	3.11	3.03	3.19
Male	3.00	2.93	3.07
**Race/color**			
White	3.09	2.98	3.20
Black	2.98	2.86	3.10
Brown	3.06	2.98	3.14
Others	2.94	2.75	3.13
**Place of death**			
Outside a hospital or health center	2.53	2.27	2.79
Inside a hospital or health center	3.07	3.02	3.12
**Capital**			
Belo Horizonte	3.39	3.28	3.50
Salvador	2.94	2.87	3.01
Natal	2.81	2.68	2.94

*Source: Ministry of Health, Mortality System Information*.

## Discussion

This study provided important information on the chain of events and contributing causes of COVID-19 adult mortality (30–69 years old) from the three Brazilian capitals cities in 2020. Three aspects were highlighted from this analysis: first, the quality of medical certification of the COVID-19 mortality; second, the importance of the process of codification of all mentions declared in the death certificate and the role of the process of investigation of the cause of death in detailing the chain of events; third, the large concentration of mentions in just a few different causes or groups of causes highlights problems in-hospital care in patients with severe COVID-19 infection, on the one hand, and the relevance of non-communicable chronic diseases as contributing conditions to the worsening of COVID-19 infection.

Regarding the first aspect, we observed that 64% of 2,921 deaths certificated with COVID-19 have the documented chain-of-event and contributing conditions. Causes mentioned as chain-of-event or contributing are in accordance with other studies (18, 19), revealing the quality of death certificates filled out by physicians in these municipalities.

The number of causes mentioned on the death certificate also indicates the quality of filling in the medical certification, reporting possible multi-morbidity, and revealing the probable access to diagnostic procedures (20, 21). On average, there were 3.04 additional conditions in death certificates with COVID-19 as UCoD. However, data showed differences among the capitals. Belo Horizonte presented a significative higher mean number of mentions than the other two capitals. These differences may be associated with the different conditions of medical care given to COVID-19 patients, the different working conditions of physicians during the pandemic, and the different cultures of medical certification of the cause of death (21). In this sense and to clarify the reasons for these differences, it is essential to carry out more in-depth research that raises the factors mentioned above and describes the process of medical certification of the cause of death by COVID-19, compared to non- COVID-19 deaths. As a background for these differences, one must consider the social and economic inequalities that distinguish the three capitals, with Belo Horizonte located in an economically more favored region than Salvador and Natal (22).

Concerning the second aspect, we must remember that the quality of cause of death statistics also depends on the coding and investigation of the cause of death process carried out by the municipality's health departments and how the guidelines from the Ministry of Health were proposed and implemented locally. In Brazil, the automatic Underlying Cause Selection System (SCB–“Seletor de Causa Básica,” in Portuguese) used in the SIM, initially code U04.9 as a marker of the COVID-19 pandemic. Subsequently and following the WHO recommendation, all death records in which the code U04.9 appeared as a pandemic marker of COVID-19 should go through the SIM update process to include the new recommended COVID-19 codes (B34.2 with the markers U07.1 or U07.2) (15, 16).

A problem with the SARS (severe acute respiratory syndrome) diagnosis mentioned in the death certificate emerged with the updated coding guidelines. Following the guidelines, the SIM would retain U04.9 (ICD-10 code for SARS) only if SARS was mentioned in the death certificate. Nevertheless, preceding these guidelines, the Ministry of Health recommended code J98.8 (other specified respiratory disorders in ICD-10) when SARS was mentioned as a single cause or accompanied by an ill-defined condition in Part I, and with no additional condition in Part II of the death certificate.

How the local coders interpreted these recommendations and guidelines and carried out a review of the codes used to identify deaths from COVID-19 is a factor that impacts the number of mentions of causes reported to the System. Thus, in the analysis of the multiple causes of death approach, it is essential to consider that, in situations such as the SARS in COVID-19 deaths, the number of codes may be higher than the mentions of diagnoses informed by the physician. In some cases, excluding diagnostic duplicity is necessary when there is more than one diagnosis meaning the same cause or more than one code for the same diagnosis.

In this study, we chose to separately present the two codes used for SARS diagnosis (U04.9 and J98.8) and emphasize the influence of coding rules and recommendations in the count of the number of diagnoses mentioned in the death certificates. A more detailed analysis to verify the co-occurrence of the two codes in the death certificates should be carried out to, eventually, correct the number of different diagnoses of causes reported by the physician.

About the investigation process, it is important to consider that the three municipalities' health departments carried out an epidemiological investigation for most of the death certificates with COVID-19 as UCoD (62.5%). However, the quality of the investigation and the information collected during this procedure also will determine the quality of cause of death statistics. Advancing this analysis to other causes would bring crucial contributions to the elucidation of the differences of number of conditions mentioned in the death certificates among the three capitals and understand the role of the codification and investigation process in improving the quality of mortality statistics.

Concerning the third aspect, more than 70% of all causes mentioned in the medical certificate in the three capitals concentrated on five conditions classified as the chain of events and six as contributing causes. As conditions that describe the chain of events that led to death by COVID-19, different respiratory complications were often mentioned as SARS, unspecified pneumonia, and acute respiratory failure. Nevertheless, the high proportion of sepsis concerns and the mention of nosocomial conditions (Y95) may be associated with the quality of the health attention in-hospital during the pandemic. An analysis of this question goes beyond the objectives of this study. This finding suggests further new studies on sepsis, such as the trend (23) and association with other causes of death through more specific multiple cause of death analysis.

As contributing conditions, there is sufficient evidence that patients with non-communicable diseases (NCD) are at higher risk of worse consequences if they contract COVID-19 (12, 24, 25). Our study also observed that the most frequent contributing conditions are hypertension, diabetes, obesity, and complications related to kidney diseases. Our results were consistent with other studies about COVID-19 associated outcomes (18). Nevertheless, it is important to note that deaths from COVID-19 are closely related to the presence of chronic conditions for the old ages (26–28). In contrast, in adults under 50, this situation is less common (19).

Considering that the situation is worse in low- and middle-income countries due to the interaction between socioecological and biological factors (29, 30), the high prevalence of NCD, the accelerated demographic transition process and the great social inequality (31) contribute to a challenging scenario in the Brazilian context of the COVID-19 pandemic.

The information obtained through this study provides essential insight on how these three capitals and other Brazilian cities need support to respond quickly and effectively to COVID-19. Also, it will be vital to ensure primary health assistance for NCD after the pandemic (24, 25, 32, 33).

However, some significant limitations of the study must be addressed. The first refers to the classification of conditions associated with COVID-19 into a chain of events and contributing. Our classification observed only the position in which the cause appeared on the death certificate. Thus, our classification depends on the accuracy with which physicians report all conditions of the morbid process and how they fill out death certificates. A second limitation is the codification process of the causes associated with COVID-19, which changed throughout 2020.

Nevertheless, the multiple-cause-of-death approach provides a starting point for the identification of other conditions that might contribute to adult mortality, considering lesser-known conditions that are not yet understood to be associated with or contribute to death from COVID-19.

## Data Availability Statement

Publicly available datasets were analyzed in this study. This data can be found at: https://datasus.saude.gov.br/transferencia-de-arquivos/.

## Ethics Statement

The analyzed data were in the public domain, preserving anonymity. Therefore, the project was approved by the Research Ethics Committee of the Federal University of Minas Gerais (COEP/UFMG) under number 4749326.

## Author Contributions

AN performed data analysis and interpretation. LI, DA, and EF proposed the grouping of causes. All authors made substantial contributions to the content, writing of this paper, conceived the study, made substantial intellectual contributions to several drafts, and approved the final version of the manuscript.

## Funding

This work was supported by Vital Strategies-GGP-CRVS Brazil Data for Health/ n. 23998 Fundep. This article is, in part, an output of the Bloomberg Philanthropies Data for Health Initiative. AN was supported in part by CNPq-Brazil (Bolsa de produtividade em pesquisa, 309944/2018-0).

## Author Disclaimer

The views expressed are not necessarily those of the Philanthropies.

## Conflict of Interest

The authors declare that the research was conducted in the absence of any commercial or financial relationships that could be construed as a potential conflict of interest.

## Publisher's Note

All claims expressed in this article are solely those of the authors and do not necessarily represent those of their affiliated organizations, or those of the publisher, the editors and the reviewers. Any product that may be evaluated in this article, or claim that may be made by its manufacturer, is not guaranteed or endorsed by the publisher.
