# Supplementary material for: Covid Adult Mortality in Brazil: An Analysis of Multiple Causes of Death
Source: Front Public Health. 2022 Jan 17;9:788932. doi: 10.3389/fpubh.2021.788932 (PMC8801696; doi:10.3389/fpubh.2021.788932)
Supplement: Supplementary file 1 [file Table_1.docx]

**Supplementary Table A - List of ICD-10 codes for cause conditions analyzed**

| **Cause conditions** | **ICD-10 Code** |
| --- | --- |
| **Chain-of-events** |  |
| Sepsis | A400-A403, A408, A409, A410-A416, A418, A419, A480, A483, A490, A491, D65, R02, R652 |
| SARS | U049 |
| Acute respiratory failure | J80, J960, J969, J983 |
| Unspecified lower respiratory infectious (Unspecified Pneumonia) | J159, J170-J173, J178, J180-J182, J187-J189, J22, P235, P236, P238, P239 |
| Other specified respiratory disorders (SARS) | J988 |
| Cardiac arrest and shock | I460-I462, I466, I468, I469, I950, I951, I958, I959, R031, R55, R570, R571, R574, R576, R578, R579 |
| Other lower respiratory infections (Pneumonia) | A481, A70, J120, J122, J123, J128, J129, J150-J152, J155, J157, J158, J160, J168, J169, J200-J209, J210, J211, J218, J219, P230-P234 |
| Other respiratory diseases | J980, J984, J985, J986, J988, J989 |
| Symptoms and signs not classified elsewhere | R000-R002, R008, R009, R010-R012, R041, R042, R048, R049, R05, R06, R070, R071-R074, R078, R079, R090, R092, R093, R098, R100-R104, R108, R109, R110, R111, R112, R119, R12, R130, R131, R139, R14R15, R160, R161, R162, R17, R170, R179, R18, R190-R196, R198, R200-R203, R208, R209, R21, R220-R224, R227, R229, R230, R231-R234, R238, R239, R250-R254, R258, R259, R260-R263, R268, R269, R270, R278, R279, R290-R299, R300, R301, R309, R32, R33, R34, R35, R36, R390-R392, R398, R399, R400-R404, R410-R414, R418, R419, R42, R430-R432, R438, R439, R440-R443, R448, R449, R450-R458, R460-R468R470, R471, R478, R479, R480-R483, R488, R489, R490-R492, R498, R499, R500, R501, R508, R509, R51R520-R522, R529, R53, R54, R560, R561, R566, R568, R569, R590, R591, R599, R600, R601, R609, R610, R611, R619, R620, R625, R627, R628, R629, R630-R633, R634, R635, R636, R638, R64 , R640, R649, R651, R680, R681, R682, R683, R688, R69, R700, R701, R71, R72, R740, R746, R748, R749, R75, R760-R762, R768, R769, R770-R772, R778, R779, R786-R789, R790, R791, R798, R799, R80, R81, R82, R83, R84, R85, R86, R87, R89, R900, R908, R91, R92, R93R940-R948, R960-R963, R98, R99 |
| Asphyxia | R090, R092, R098 |
| Pulmonary Embolism | I260, I269 |
| External Causes - other factors | Y900, Y901, Y902, Y903, Y904, Y905, Y906, Y907, Y908, Y909, Y91, Y910, Y911, Y912, Y913, Y919, Y92, Y920, Y921, Y922, Y923, Y924, Y925, Y926, Y927, Y928, Y929, Y93, Y930, Y931, Y932, Y933, Y934, Y935, Y936, Y937, Y938, Y939, Y94, Y95, Y96, Y97, Y98, Y980, Y99, Y990, Y991, Y992, Y998, Y999 |
| Acidosis | E872 |
| Upper respiratory infection unspecified | J029, J039, J069 |
| Urinary tract infections | N10, N110, N111, N118, N119, N12, N151, N158, N159, N160-N165, N168, N300-N304, N308, N309, N340-N343, N390-N392 |
| Pulmonary Edema | J81 |
| Hyperkalemia | E875 |
|  |  |
|  |  |
| **Cause conditions** | **ICD-10 Code** |
| **Contributing conditions** |  |
| Hypertension | I10, I150-I152, I158, I159, R030, R040 |
| Diabetes, unspecified type | E120, E121, E123-E129, E130, E131, E133-E139, E140, E141, E143-E149, R730, R739 |
| Renal Failure | N170-N172, N178, N179, N19 |
| Obesity | E65, E660, E662, E663, E668, E669 |
| Other Chronic kidney diseases | D631, N180-N186, N188, N189 |
| Diabetes mellitus type 2 | E110, E111, E113-E119 |
| Heart failure | I500-I504, I508, I509, I517 |
| Ischemic heart disease | I200, I201, I208, I209, I210-I214, I216, I217, I219, I220-I222, I228, I229, I230-I238, I240, I241, I248, I249, I250-I259 |
| Hemodialysis | Y841 |
| Chronic obstructive pulmonary disease | J410, J411, J418, J42, J430-J432, J438, J439, J440, J441, J448, J449 |
| Chronic kidney disease due to glomerulonephritis | N030-N039, N040-N049, N050-N059, N060-N069 |
| Other mental disorders | 063, F064, F072, F09, F090, F099, F17, F170, F171, F172, F173, F174, F175, F176, F177, F178, F179, F30, F300, F301, F302, F303, F304, F308, F309, F31, F310, F311, F312, F313, F314, F315, F316, F317, F318, F319, F32, F320, F321, F322, F323, F324, F325, F328, F329, F33, F330, F331, F332, F333, F334, F338, F339, F34, F340, F341, F348, F349, F35, F36, F37, F38, F380, F381, F388, F39, F40, F400, F401, F402, F408, F409, F41, F410, F411, F412, F413, F418, F419, F42, F420, F421, F422, F428, F429, F43, F430, F431, F432, F438, F439, F44, F440, F441, F442, F443, F444, F445, F446, F447, F448, F449, F45, F450, F451, F452, F453, F454, F458, F459, F46, F47, F48, F480, F481, F482, F488, F489, F49, F51, F510, F511, F512, F513, F514, F515, F518, F519, F52, F520, F521, F522, F523, F524, F525, F526, F527, F528, F529, F53, F530, F531, F538, F539, F54, F55, F550, F551, F552, F553, F554, F558, F559, F56, F57, F58, F59, F60, F600, F601, F602, F603, F604, F605, F606, F607, F608, F609, F61, F62, F620, F621, F628, F629, F63, F630, F631, F632, F633, F638, F639, F64, F640, F641, F642, F648, F649, F65, F650, F651, F652, F653, F654, F655, F656, F658, F659, F66, F660, F661, F662, F668, F669, F67, F68, F680, F681, F688, F69, F690, F70, F700, F701, F708, F709, F71, F710, F711, F718, F719, F72, F720, F721, F728, F729, F73, F730, F731, F738, F739, F74, F75, F76, F77, F78, F780, F781, F788, F789, F79, F790, F791, F798, F799, F80, F800, F801, F802, F803, F804, F808, F809, F81, F810, F811, F812, F813, F818, F819, F82, F820, F83, F84, F840, F841, F842, F843, F844, F845, F848, F849, F85, F86, F87, F88, F89, F890, F90, F900, F901, F902, F908, F909, F91, F910, F911, F912, F913, F918, F919, F92, F920, F928, F929, F93, F930, F931, F932, F933, F938, F939, F94, F940, F941, F942, F948, F949, F95, F950, F951, F952, F958, F959, F96, F97, F98, F980, F981, F982, F983, F984, F985, F986, F988, F989, F99, F990 |
| Stroke, unspecified | I64, I679, I688, I694, I698, I699 |
| Alcohol use disorders | F100-F109, G312, G721, P043, Q860, R780, X450-X459, X650-X659, Y15 |
|  |  |
|  |  |
|  |  |
|  |  |
|  |  |
| **Cause conditions** | **ICD-10 Code** |
| **Contributing conditions** |  |
| Adverse effects of medical treatment | Y40, Y41, Y42, Y43, Y44, Y450-Y455, Y458, Y459, Y460-Y468, Y470-Y475, Y478, Y479, Y480-Y485, Y49, Y500-Y502, Y508, Y509, Y51, Y52, Y53, Y54, Y550-Y557, Y559, Y56, Y57, Y580-Y586, Y588, Y589, Y590-Y593, Y598, Y599, Y60, Y61, Y620-Y626, Y628, Y629, Y630-Y636, Y638, Y639, Y640, Y641, Y648, Y649, Y650-Y655, Y658, Y66, Y69, Y700-Y703, Y708, Y710-Y713, Y718, Y720-Y723, Y728, Y730-Y733, Y738, Y740-Y743, Y748, Y750-Y753, Y758, Y760-Y763, Y768, Y769, Y770-Y773, Y778, Y780-Y783, Y788, Y790-Y793, Y798, Y800-Y803, Y808, Y810-Y813, Y818, Y820-Y823, Y828, Y829, Y830-Y836, Y838, Y839, Y840-Y849, Y880-Y883 |
| Asthma | J450-J455, J458, J459, J46 |
| Cirrhosis and other chronic liver diseases | B180-B182, B188, B189, I850, I851, I859, I982, K700-K703, K717, K740-K749, K752, K754, K758, K759, K760-K762, K764-K769, K778 |
| Tracheal, bronchus, and lung cancer | C33, C340-C344, C347-C349, D021-D023, D142, D143, D381 |
| Cancers, unspecified site | C764, C765, C767-C769, C773, C774, C778, C779, C792-C799, C80, C800, C801, C802, C809, C97, D097, D099, D360, D369, D487, D489, D498, D499, |
| Breast cancer | C500-C509, D050, D051, D057, D058, D059, D24, D486, D493 |
| Ischemic stroke | G450-G454, G458, G459, G460-G468, I630-I639, I650-I653, I658, I659, I660-I664, I668, I669, I672, I673, I675, I676, I693, I678 |
| Atrial fibrillation and flutter | I48, I480-I484, I489 |
| Heart Diseases, unspecified | I518, I519 |
| Hypertensive heart disease | I110, I112, I116, I119 |
| Hypothyroidism | E030, E031, E033-E035, E038, E039, E040-E042, E048, E049, E060-E063, E065, E069, E070, E071 |
| Other endocrine, metabolic, blood, immune diseases | D66 , D67, D680-D686, D688, D689, D690-D694, D696, D698, D70, D71, D720, D721, D728, D729, D730-D735, D738, D739, D740, D748, D749, D750-D752, D758, D760-D763, D77 , D868, D890-D893, E161-E164, E168, E169, E200, E201, E208, E209, E210-E215, E220-E222, E228, E229, E230, E232, E233, E236, E237, E240, E241, E243, E244, E248, E249, E250, E258, E259, E260, E261, E268, E269, E270-E272, E274, E275, E278, E279, E280, E281, E283, E288, E289, E290, E291, E298, E299, E300, E301, E308, E309, E310-E312, E318, E319, E320, E321, E328, E329, E340-E345, E348, E670-E673, E678, E68, E700-E705, E708, E709, E710-E715, E720-E725, E728, E729, E730, E731, E738, E739, E740-E744, E748, E749, E750-E755, E756, E760-E763, E768, E769, E770, E771, E778, E779, E790-E792, E798, E799, E800-E807, E830-E839, E850-E852, E880-E889 |
| Alzheimer disease and other dementias | F000-F002, F009, F010-F013, F015, F018, F019, F020-F024, F028, F03, G300, G301, G308, G309, G310, G311, G318, G319 |
| Epilepsy | G400-G409, G410-G412, G418, G419 |
| Pyoderma | A460, A660-A669, A670-A673, A677, A679, I891, I898, L00, L010, L011, L020-L026, L028, L029, L040-L043, L048, L049, L050, L059, L080, L081, L088, L089, L88, L97, L980-L984 |
| Schizophrenia | F062, F200-F209, F21, F220, F228, F229, F230-F233, F238, F239, F250-F252, F258, F259, F28, F29 |
| Colon and rectum cancer | C180-C189, C19, C20, C210-C212, C218, C219, D010-D013, D120-D129, D373-D375 |
| Peripheral artery disease | I702-I708, I730, I731, I738, I739 |
|  |  |
|  |  |
| **Cause conditions** | **ICD-10 Code** |
| **Conditions not classified (most frequent)** |  |
| Gastrointestinal Bleeding | K920-K922 |
| Other arrhythmias | I490-I495, I498, I499 |
| Hepatic Failure | K720, K721, K729 |
| Injuries | G443, G913, R58, S000-S005, S007-S009, S010-S015, S017-S019, S02, S030-S035, S038, S039, S04, S05, S06, S070-S071, S078, S079, S080, S081, S088, S089, S090-S093, S097-S099, S100, S101, S107-S109, S110-S112, S117-S119, S12, S130-S136, S138, S139, S140-S146, S148, S149, S150-S153, S157-S159, S16, S170, S178, S179, S18, S197, S198, S199, S200-S204, S207-S209, S210-S214, S217-S219, S220-S225, S228, S229, S230-S235, S238, S239, S240-S246, S248, S249, S250-S255, S257-S259, S260, S261, S268, S269, S27, S280-S282, S290, S297-S299, S300-S303, S307-S309, S310-S315, S317, S318, S320-S325, S327-S329, S33, S340-S346, S348, S430-S436, S349, S350-S355, S357-S359, S36, S37, S380-S383, S390, S396-S399, S400, S402, S407-S409, S410, S411, S417, S418, S420-S424, S427-S429, S437-S439, S440-S445, S447-S449, S450-S453, S457-S459, S460-S463, S467-S469, S47, S480, S481, S489, S490, S491, S497-S499, S500, S501, S503, S507-S509, S510, S517-S519, S52, S530-S534, S537, S540-S543, S547-S549, S550-S552, S557-S559, S560-S565, S567-S569, S570, S578, S579, S580, S581, S589, S590-S592, S597-S599, S600-S605, S607-S609, S610-S615, S617-S619, S62, S630-S637, S639, S640-S644, S647-S649, S650-S655, S657-S659, S66, S670-S674, S678, S679, S68, S697-S699, S700-S703, S707-S709, S710, S711, S717, S718, S720-S724, S727-S729, S730, S731, S740-S742, S747-S749, S750-S752, S757-S759, S760-S764, S767-S769, S770-S772, S780, S781, S789, S790, S791, S797, S798, S799, S800-S802, S807-S809, S810, S817-S819, S82, S83, S840-S842, S847-S849, S850-S855, S857-S859, S860-S863, S867-S869, S870, S878, S880, S881, S889, S890-S893, S897-S899, S900-S905, S90, S910-S913, S917, S92, S930-S936, S940-S942, S943, S947-S949, S950-S952, S957-S959, S960-S962, S967-S969, S970, S971, S978, S980-S984, S989, S997-S999, T000-T003, T006, T008, T009, T010-T013, T016, T018, T019, T02, T030-T034, T038, T039, T040-T044, T047-T049, T050-T056, T058, T059, T060-T065, T068, T07, T08, T090-T096, T098, T099, T10, T110-T116, T118, T119, T12, T130-T136, T138, T139, T140-T149, T150, T151, T158, T159, T16, T170-T175, T178, T179, T180-T185, T188, T189, T190-T194, T198, T199, T200-T207, T210-T217, T219, T220-T227, T230-T237, T240-T247, T250-T257, T26, T270-T277, T28, T290-T297, T300-T307, T310-T319, T32, T33, T34, T350-T357, T36, T37, T38, T390-T394, T398, T399, T40, T410-T415, T420-T428, T430-T436, T438, T439, T44, T45, T46, T47, T480-T487, T489, T49, T50, T510-T513, T518, T519, T520-T524, T528, T529, T530-T537, T539, T540-T543, T549, T55, T56, T570-T573, T578, T579, T58, T59, T600-T604, T608, T609, T610-T612, T617-T619, T620-T622, T628, T629, T63, T64, T650-T656, T658, T659, T66, T670, T68, T690, T691, T698, T699, T700-T704, T708, T709, T71, T730-T733, T738, T739, T740-T744, T748, T749, T750-T754, T758, T76, T780-T784, T788, T789, T79, T800-T806, T808, T809, T81, T82, T83, T84, T85, T860-T865, T868, T869, T870-T876, T878, T879, T88, T900-T905, T908, T909, T910-T915, T918, T919, T920-T926, T928, T929, T930-T936, T938, T939, T940, T941, T950-T954, T958, T959, T96, T97, T980-T983, W47, W48, W63, W71, W72 , W76, W82, W95, W96, W97, W98, X07, X400-X402, X404-X409, X41, X42, X43, X44, X49, X56, X59, Y10, Y11, Y12, Y13, Y14, Y16, Y17, Y18, Y19, Y20, Y21, Y22, Y23, Y24, Y250-Y252, Y254-Y259, Y26, Y27, Y28, Y290, Y30, Y31, Y32, Y33, Y34, Y86, Y872, Y899 |
