# Supplementary material for: Covid Adult Mortality in Brazil: An Analysis of Multiple Causes of Death
Source: Front Public Health. 2022 Jan 17;9:788932. doi: 10.3389/fpubh.2021.788932 (PMC8801696; doi:10.3389/fpubh.2021.788932)
Supplement: Supplementary file 2 [file Table_2.docx]

**Supplementary Table B - Conditions listed in death certificates by position in relation to COVID-19 diagnosis, at ages 30–69 years, in three Brazilian capitals, 2020**

| **Cause conditions** | **Number of mentions** | | **Position in relation to COVID-19 diagnosis** | | **% of COVID-19 deaths (n=2843)** |
| --- | --- | --- | --- | --- | --- |
|  | **n** | **%** | **Above** | **Below** |  |
| **Chain-of-events** |  |  |  |  |  |
| Sepsis | 950 | 10,8 | 96,1 | 3,9 | 33,4 |
| SARS | 909 | 10,3 | 99,4 | 0,6 | 32,0 |
| Acute respiratory failure | 907 | 10,3 | 96,1 | 3,9 | 31,9 |
| Unspecified lower respiratory infectious (Unspecified Pneumonia) | 571 | 6,5 | 94,2 | 5,8 | 20,1 |
| Other specified respiratory disorders (SARS) | 400 | 4,5 | 96,5 | 3,5 | 14,1 |
| Cardiac arrest and shock | 257 | 2,9 | 97,7 | 2,3 | 9,0 |
| Other lower respiratory infections (Pneumonia) | 141 | 1,6 | 95,0 | 5,0 | 5,0 |
| Other respiratory diseases | 105 | 1,2 | 94,3 | 5,7 | 3,7 |
| Symptoms and signs not classified elsewhere | 98 | 1,0 | 96,7 | 3,3 | 3,4 |
| Asphyxia | 89 | 1,0 | 94,4 | 5,6 | 3,1 |
| Pulmonary Embolism | 61 | 0,7 | 77,0 | 23,0 | 2,1 |
| External Causes - other factors | 30 | 0,3 | 86,7 | 13,3 | 1,1 |
| Acidosis | 27 | 0,3 | 74,1 | 25,9 | 0,9 |
| Upper respiratory infection unspecified | 26 | 0,3 | 100,0 | 0,0 | 0,9 |
| Urinary tract infections | 22 | 0,2 | 63,6 | 36,4 | 0,8 |
| Pulmonary Edema | 19 | 0,2 | 94,7 | 5,3 | 0,7 |
| Hyperkalemia | 18 | 0,2 | 72,2 | 27,8 | 0,6 |
| **Contributing conditions** |  |  |  |  |  |
| Hypertension | 950 | 10,8 | 96,1 | 3,9 | 33,4 |
| Diabetes, unspecified type | 909 | 10,3 | 99,4 | 0,6 | 32,0 |
| Renal Failure | 907 | 10,3 | 96,1 | 3,9 | 31,9 |
| Obesity | 571 | 6,5 | 94,2 | 5,8 | 20,1 |
| Other Chronic kidney diseases | 400 | 4,5 | 96,5 | 3,5 | 14,1 |
| Diabetes mellitus type 2 | 257 | 2,9 | 97,7 | 2,3 | 9,0 |
| Heart failure | 141 | 1,6 | 95,0 | 5,0 | 5,0 |
| Ischemic heart disease | 105 | 1,2 | 94,3 | 5,7 | 3,7 |
| Hemodialysis | 98 | 1,0 | 96,7 | 3,3 | 3,4 |
| Chronic obstructive pulmonary disease | 89 | 1,0 | 94,4 | 5,6 | 3,1 |
| Chronic kidney disease due to glomerulonephritis | 61 | 0,7 | 77,0 | 23,0 | 2,1 |
| Other mental disorders | 30 | 0,3 | 86,7 | 13,3 | 1,1 |
| Stroke, unspecified | 27 | 0,3 | 74,1 | 25,9 | 0,9 |
| Alcohol use disorders | 26 | 0,3 | 100,0 | 0,0 | 0,9 |
| Adverse effects of medical treatment | 22 | 0,2 | 63,6 | 36,4 | 0,8 |
| Asthma | 19 | 0,2 | 94,7 | 5,3 | 0,7 |
| Cirrhosis and other chronic liver diseases | 18 | 0,2 | 72,2 | 27,8 | 0,6 |
| Tracheal, bronchus, and lung cancer | 950 | 10,8 | 96,1 | 3,9 | 33,4 |
| Cancers, unspecified site | 909 | 10,3 | 99,4 | 0,6 | 32,0 |
| Breast cancer | 907 | 10,3 | 96,1 | 3,9 | 31,9 |
| Ischemic stroke | 571 | 6,5 | 94,2 | 5,8 | 20,1 |
| Atrial fibrillation and flutter | 400 | 4,5 | 96,5 | 3,5 | 14,1 |
| Heart Diseases, unspecified | 257 | 2,9 | 97,7 | 2,3 | 9,0 |
| Hypertensive heart disease | 141 | 1,6 | 95,0 | 5,0 | 5,0 |
| Hypothyroidism | 105 | 1,2 | 94,3 | 5,7 | 3,7 |
| Other endocrine, metabolic, blood, immune diseases | 98 | 1,0 | 96,7 | 3,3 | 3,4 |
| Alzheimer disease and other dementias | 89 | 1,0 | 94,4 | 5,6 | 3,1 |
| Epilepsy | 61 | 0,7 | 77,0 | 23,0 | 2,1 |
| Pyoderma | 30 | 0,3 | 86,7 | 13,3 | 1,1 |
| Schizophrenia | 27 | 0,3 | 74,1 | 25,9 | 0,9 |
| Colon and rectum cancer | 26 | 0,3 | 100,0 | 0,0 | 0,9 |
| Peripheral artery disease | 22 | 0,2 | 63,6 | 36,4 | 0,8 |
|  |  |  |  |  |  |
| **Cause conditions** |  |  |  |  |  |
| **Conditions not classified (most frequent)** |  |  |  |  |  |
| Injuries | 24 | 0,3 | 58,3 | 41,7 | 0,8 |
| Gastrointestinal Bleeding | 22 | 0,2 | 59,1 | 40,9 | 0,8 |
| Other arrhythmias | 19 | 0,2 | 57,9 | 42,1 | 0,7 |
| Hepatic Failure | 19 | 0,2 | 47,4 | 52,6 | 0,7 |
